# Contrasting Measures of Cerebrovascular Reactivity Between MRI and Doppler: A Cross-Sectional Study of Younger and Older Healthy Individuals

**DOI:** 10.3389/fphys.2021.656746

**Published:** 2021-04-12

**Authors:** Claire V. Burley, Susan T. Francis, Kate N. Thomas, Anna C. Whittaker, Samuel J. E. Lucas, Karen J. Mullinger

**Affiliations:** ^1^School of Sport, Exercise and Rehabilitation Sciences, University of Birmingham, Birmingham, United Kingdom; ^2^Centre for Human Brain Health, University of Birmingham, Birmingham, United Kingdom; ^3^Dementia Centre for Research Collaboration, School of Psychiatry, University of New South Wales, Sydney, NSW, Australia; ^4^Sir Peter Mansfield Imaging Centre, School of Physics and Astronomy, University of Nottingham, Nottingham, United Kingdom; ^5^Department of Surgical Sciences, Dunedin School of Medicine, University of Otago, Dunedin, New Zealand; ^6^Faculty of Health Sciences and Sport, University of Stirling, Stirling, United Kingdom; ^7^School of Psychology, University of Birmingham, Birmingham, United Kingdom

**Keywords:** cerebrovascular reactivity, transcranial Doppler, magnetic resonance imaging, ageing, brain vascular health, cerebral blood flow

## Abstract

Cerebrovascular reactivity (CVR) is used as an outcome measure of brain health. Traditionally, lower CVR is associated with ageing, poor fitness and brain-related conditions (e.g. stroke, dementia). Indeed, CVR is suggested as a biomarker for disease risk. However, recent findings report conflicting associations between ageing or fitness and CVR measures. Inconsistent findings may relate to different neuroimaging modalities used, which include transcranial Doppler (TCD) and blood-oxygen-level-dependant (BOLD) contrast magnetic resonance imaging (MRI). We assessed the relationship between CVR metrics derived from two common imaging modalities, TCD and BOLD MRI, within the same individuals and with expected significant differences (i.e., younger vs. older) to maximise the expected spread in measures. We conducted two serial studies using TCD- and MRI-derived measures of CVR (via inspired 5% CO_2_ in air). Study 1 compared 20 younger (24 ± 7 years) with 15 older (66 ± 7 years) participants, Study 2 compared 10 younger (22 ± 2 years) with 10 older (72 ± 4 years) participants. Combining the main measures across studies, no significant correlation (*r* = 0.15, *p* = 0.36) was observed between individual participant TCD- and BOLD-CVR measures. Further, these measures showed differential effects between age groups; with TCD-CVR higher in the older compared to younger group (4 ± 1 vs. 3 ± 1 %MCAv/mmHg P_*ET*_CO_2_; *p* < 0.05, *Hedges’ g* = 0.75), whereas BOLD-CVR showed no difference (*p* = 0.104, *Hedges’ g* = 0.38). In Study 2 additional measures were obtained to understand the origin of the discrepancy: phase contrast angiography (PCA) MRI of the middle cerebral artery, showed a significantly lower blood flow (but not velocity) CVR response in older compared with younger participants (*p* > 0.05, *Hedges’ g* = 1.08). The PCA CVR metrics did not significantly correlate with the BOLD- or TCD-CVR measures. The differing CVR observations between imaging modalities were despite expected, correlated (*r* = 0.62–0.82), age-related differences in resting CBF measures across modalities. Taken together, findings across both studies show no clear relationship between TCD- and BOLD-CVR measures. We hypothesize that CVR differences between imaging modalities are in part due to the aspects of the vascular tree that are assessed (TCD:arteries; BOLD:venules/veins). Further work is needed to understand the between-modality CVR response differences, but caution is needed when comparing CVR metrics derived from different imaging modalities.

## Introduction

The methods chosen through which to assess brain health and individual vascular disease risk (e.g., stroke), as well as the efficacy of approaches to improve brain health are important areas of research to improve the healthspan of individuals (i.e., the period of one’s life that is healthy). One method which is becoming established for the assessment of brain health and a potential biomarker for disease risk is cerebrovascular reactivity (CVR) (e.g., Kleiser and Widder, 1992; Silvestrini et al., 2000; Portegies et al., 2014; Smeeing et al., 2016; Juttukonda and Donahue, 2019; Sur et al., 2020). CVR is the reactivity of blood vessels in the brain to an external isometabolic (i.e., no change in brain metabolism) stimulus. The idea is to assess how cerebral blood flow (CBF) changes when a stimulus is presented. To disassociate the vascular response to changes in neuronal activity between cohorts or time points of interest, the stimulus of choice is often carbon dioxide (CO_2_), a potent vasodilator of the cerebrovasculature (Willie et al., 2014). Furthermore, it is thought to cause only a small change in the cerebral metabolic rate of oxygen consumption (CMRO_2_) (Peng et al., 2017), and is readily available and easy to administer. Following a period of resting (baseline) CBF measures, CBF is then assessed whilst the partial pressure of CO_2_ in the arteries (PaCO_2_) is altered either via increases in the inspired CO_2_ concentration (i.e., hypercapnia) to elevate CBF, or from hyperventilation to lower PaCO_2_ (i.e., hypocapnia) and reduce CBF. The changes in PaCO_2_ are quantified via measures of CO_2_ in the expired breath (i.e., end-tidal CO_2_ (P_*ET*_CO_2_)). By taking the ratio of the relative change in CBF to the change in P_*ET*_CO_2_, the relative CVR is found. This CVR measure therefore gives a metric of the regulatory function of the brain’s vasculature (Fierstra et al., 2013).

There are a range of methods to measure CBF, and hence CVR, either directly or indirectly. Positron emission tomography (PET) and single photon emission computer tomography (SPECT) (Juttukonda and Donahue, 2019) both can provide measures of CBF, but require the use of radioactive isotopes and therefore are undesirable to use unless clinically beneficial. Non-ionising methods of measuring CVR are primarily through the use of Doppler ultrasound or Magnetic Resonance Imaging (MRI) (Juttukonda and Donahue, 2019). Transcranial Doppler (TCD) measures blood velocity within an artery [often the middle cerebral artery (MCA)] as a proxy for CBF. For the use of velocity as a proxy for flow to be valid, it assumes that there is no change in the cross-sectional area of the artery on stimulus administration, and that this effect is consistent between the compared cohorts. However, it has recently been shown that there are likely to be changes in blood vessel diameter with gas challenge (Verbree et al., 2014; Coverdale et al., 2017), as well as an increase in blood velocity. Doppler ultrasound of the extracranial arteries (internal carotid and vertebral arteries) is being used increasingly commonly to overcome this limitation as both diameter and velocity can be measured simultaneously for the calculation of flow (Thomas et al., 2015); however, this method is not yet as widely used as TCD for CVR due to it being somewhat more technically demanding to perform. Nevertheless, despite its velocity-based limitation, TCD is still frequently used to assess CVR due to its low cost, portability and the fact that any cohort can be imaged with this technique (i.e., there are no safety implications unlike MRI).

MRI-based CVR measures have most commonly been performed by utilising the blood oxygen level dependant (BOLD) signal due to its high signal-to-noise ratio (SNR) and easy accessibility to MRI sequences that can measure this signal. The BOLD signal is increased in the presence of reduced concentration of deoxyhaemoglobin (Ogawa et al., 1990) and reflects the complex balance of CBF, CMRO_2_ and blood volume that together alter the concentration of deoxyhaemoglobin. Since the BOLD signal is sensitive to changes in the concentration of deoxyhaemoglobin, the signal measured is from the venous side of the vascular tree and is primarily thought to originate in the venules (Ogawa et al., 1990; Gagnon et al., 2015). The BOLD signal therefore provides an indirect measure of changes in CBF in the venules. The BOLD signal is used extensively to examine brain vascular response to an external stimulus, including to induced changes in PaCO_2_ as described above, with an assumption that such a hypercapnic challenge results in no change in neuronal activity and therefore CMRO_2_ (Febo, 2011; Buxton, 2013; Peng et al., 2017). In addition to the BOLD approach, other MRI techniques are also used to assess CVR. These include arterial spin labelling (ASL), which measures CBF in the grey matter tissue (Alsop et al., 2015), and phase contrast angiography (PCA), which measures blood velocity and flow in large vessels such as the MCA. However, ASL has poor SNR relative to BOLD, and PCA has a very poor time resolution (in the order of a minute per measure). Therefore, BOLD CVR measures are the most commonly used MRI method, although the least direct of the MRI options for measuring the signal of interest, CBF.

Traditionally, higher CVR is associated with higher aerobic fitness (Bailey et al., 2013; Barnes et al., 2013), while a lower CVR is associated with natural ageing (Bakker et al., 2004; Lu et al., 2011; Gauthier et al., 2013, 2015; Bhogal et al., 2016; Peng et al., 2018; Miller et al., 2019) and brain-related conditions including dementia (den Abeelen et al., 2014) and stroke (Markus and Cullinane, 2001). Therefore, a higher CVR has been perceived as indexing a healthier brain. However, recent findings challenge some of these observations and assumptions. For example, blunted CVR was reported in a group of Masters athletes relative to sedentary age-matched controls (Thomas et al., 2013), and a recent study showed a similar negative relationship between CVR and fitness (Furby et al., 2020). This finding has also been shown in studies of older adults, where higher cardiorespiratory fitness was negatively related to CVR (DuBose et al., 2016; Intzandt et al., 2020). In fact, a number of studies report findings that conflict with the simplistic age-related CVR relationship as well (Kastrup et al., 1998; Galvin et al., 2010; Murrell et al., 2013). Thus, while simplistic relationships between CVR and age and/or fitness are commonly portrayed, there are a lot of inconsistencies within the literature around this topic.

A plausible explanation for the discrepancy between the CVR changes reported in different studies is the imaging modality used. Indeed, while the reduced CVR with greater fitness has been reported in studies using MRI, primarily using BOLD contrast CVR (Thomas et al., 2013; DuBose et al., 2016; Furby et al., 2020), it is Doppler-based imaging methods that show an increase in CVR with higher fitness levels (Bailey et al., 2013; Barnes et al., 2013; Murrell et al., 2013). Whilst the majority of studies show a decrease in CVR with age, the studies where no change or an increase has been reported are mostly using Doppler as the imaging modality (Kastrup et al., 1998; Galvin et al., 2010; Murrell et al., 2013); notwithstanding the sex-differences with ageing that Kastrup and colleagues reported in their TCD-based study (Kastrup et al., 1998) and a PET-based study that showed no ageing effect on CVR (Ito et al., 2002).

Comparison of CVR findings between studies using either TCD- or BOLD MRI-derived measures are often made to help the interpretation of a new result with previous studies on physiologically relevant cohorts. The assumption in such comparisons is that all CVR measures are reflecting the same underlying physiology often without consideration of the imaging method used. However, direct comparison of TCD and BOLD MRI techniques for measuring CVR has not been made. The expected relationship between CVR measures across TCD and BOLD MRI is not immediately clear due to the different aspects of the vascular tree they are measuring from (TCD: arteries; BOLD: venules and veins), and the fact both are providing indirect measures of CBF, as described above. If CVR is to be a measure of brain health and a biomarker for disease prognosis and/or diagnosis (as highlighted above), then it is vital that we understand the precise relationship between these different approaches that all collectively refer to CVR. Thus, establishing if these different methods of determining CVR all reflect the same underlying brain health status is crucial, since if they provide different information and outcomes, they should not all be referred to as the same thing; i.e., the single term CVR.

Here we aim to perform a direct comparison of CVR derived from TCD and BOLD MRI measures, two common methods used to assess CVR. Importantly, we do this in the same group of participants with the same experimental setup and CO_2_ delivery method. The same experimental set-up was important as aspects of experimental set-up can also affect the determined CVR outcome, such as CO_2_ stimulus concentration and duration, as well as where the steady-state time point is taken from Burley et al. (2020). This work combines two studies. The first study aimed to compare age and fitness effects on CVR within and between imaging modalities, recruiting both younger and older adults that were either fit or unfit. Preliminary results of study one indicated no relationship between TCD and BOLD MRI CVR measures which required further investigation. The aim of the second study was to compare CVR outcomes between imaging modalities for the two groups that were expected to be the most different (younger fit vs. sedentary older adults). In study two, PCA MRI was employed to provide a mid-stage measure of CVR with the aim of furthering understanding of differences in CVR calculated with TCD and BOLD MRI. We hypothesised that CVR derived from TCD and BOLD MRI would not correlate across age and fitness groups, owing to the different origins of the signal measured; while measured metrics that were more direct measures of CBF (i.e., MCA velocity measured by TCD vs. PCA MRI) would show better agreement. This work will help inform the comparison and interpretation of existing studies using either of the imaging modalities, as well as how CVR can be used as a biomarker in the future.

## Methods

For both studies, ethical approval was obtained from the Science, Technology, Engineering and Mathematics Ethical Review Committee, University of Birmingham and conformed to the Declaration of Helsinki (project code: ERN_14-1423). Prior to participation, a detailed verbal and written explanation of the study was provided, and written informed consent was obtained.

## Study 1

### Participants

Thirty-five healthy volunteers in two age groups participated: 20 younger participants, mean age 24 ± 7 years and 15 older participants, mean age 66 ± 7 years. Participants were excluded if they had any neurological or psychiatric conditions or if any abnormalities were revealed from a 12-lead electrocardiogram (ECG). Participants were not taking any medication (with the exception of oral contraception in females) and had no history of cardiovascular, cerebrovascular or respiratory disease.

Groups were further divided into fit and unfit groups, as determined by performance on a maximum oxygen consumption (V̇O_2_max) fitness test (see below for details). The partitioning of fitness for each age group was as follows: for younger participants, a V̇O_2_max > 45 mL^⋅^min^–1^^⋅^kg^–1^ placed them in the fit group; for the older participants, a V̇O_2_max > 25 mL^⋅^min^–1^^⋅^kg^–1^ placed them in the fit group. Remaining participants were placed in the associated unfit groups. Partitioning values were based on normative data, which includes the well-established decline in cardiorespiratory fitness across the lifespan (Heyward, 1998; Riebe et al., 2018).

### Overview

Participants for this study completed five visits in total. The first visit included general health screening, MRI safety screening, fitness questionnaires, and an electrocardiogram (if over 50 years of age, see Supplementary Methods). After inclusion, the second visit comprised the aerobic fitness test on a treadmill or stationary bike to determine the maximal oxygen consumption (V̇O_2_max) of each participant (see Supplementary Methods). The third visit was a familiarization of the CBF measures using TCD and gas challenges (not reported). After satisfactory completion of familiarisation trials (i.e., no adverse reactions to breathing the CO_2_ stimulus were experienced by the participant and adequate Doppler signals were identified), participants were invited back for the two full testing sessions (visits four and five), one where MCAv was measured using TCD and one where BOLD and ASL signals were measured using MRI to give different direct and indirect measures of brain blood flow at rest and during the CO_2_ gas challenge (i.e., CVR test). The order of these final two visits was randomised and counter-balanced between participants, using a computer random number generator, to avoid order effects. For all visits, participants were asked to avoid vigorous exercise and alcohol for 24 h, caffeine for 12 h and heavy meals for 4 h prior to study participation.

### Cerebrovascular Reactivity Test

**FIGURE 1:**
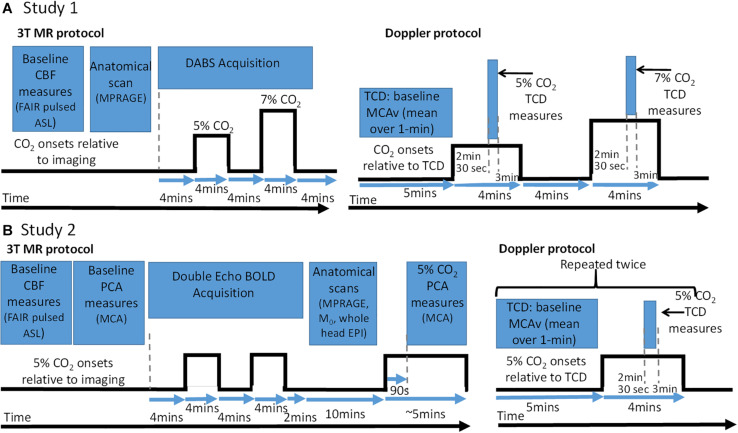
Baseline CBF and cerebrovascular reactivity (CVR) test protocol schematic and accompanying measure timing information for MRI and TCD sessions. **(A)** Protocols for Study 1. **(B)** Protocols for Study 2. Note for TCD data – data were recorded over whole gas challenge but only data during highlighted time windows was used to calculate CBF and CVR metrics. CO_2_ onsets: are when the CO_2_ gas (5 or 7%) were presented relative all the imaging data which were acquired. CBF, cerebral blood flow; PCA, phase contrast angiography; TCD, transcranial Doppler; MCA, middle cerebral artery; FAIR, flow-sensitive alternating inversion recovery; ASL, arterial spin labelling; BOLD, blood oxygen level dependant; EPI, echo planar imaging; MPRAGE, magnetisation prepared rapid gradient echo.

Participants were asked to lie in supine position, relax and breathe as naturally as possible. The CVR test was performed after a resting period of at least 20 min. It consisted of a sequential pattern of 4-min period of breathing room air, 4 min of breathing a 5% CO_2_ stimulus, 4 min of room air, 4 min of 7% CO_2_ stimulus, 4 min of room air as shown in Figure 1A.

To ensure mouth breathing only, participants wore a nose peg whilst breathing through a mouthpiece connected to a 3-way valve (2700 series: Hans Ruldolph Inc., United States) and an inhalation and exhalation tube. The inhalation tube was connected to one of two open-circuit Douglas bags (containing 5% CO_2_ or 7% CO_2_ gas in dry air) or left open to room air. Within this circuit for the TCD session only, was a heated pneumotachograph (3813 Series, Hans Rudolph Inc., United States); however, due to the non-MRI compatibility of any available spirometer, ventilation volume and rate was not recorded during the MRI session. Switching the Douglas bag valves from ambient room air to the 5 and 7% CO_2_ mixture was done by a researcher sitting beside the participant for both TCD and MRI sessions.

During both visits, fractional changes in inspired and expired oxygen (O_2_) and carbon dioxide (CO_2_) were measured via a sample line inserted into the mouthpiece and connected to a fast-responding gas analyser (ML206, ADInstruments Ltd, New Zealand), with the flow rate at maximum (200 mL^⋅^min^–1^). O_2_ and CO_2_ data were acquired continuously at a sample rate of 1 kHz via an analogue-to-digital converter (PowerLab, ADInstruments) interfaced with a computer, displayed in real time and stored for offline analysis using commercially available software (LabChart v7.3.5, ADInstruments). Known gas concentrations were used to calibrate the gas analyser prior to every testing session.

In addition, for the TCD session only, beat-by-beat blood pressure (BP) was measured using photoplethysmography via a finger cuff placed on the middle finger of the left hand (Portapres, Finapres Medical Systems BV, the Netherlands). A 3-lead electrocardiogram (ECG) was used in the TCD session to continuously measure heart rate. During the MRI visit, cardiac and respiratory cycles were simultaneously recorded using the vector cardiogram (VCG; Philips Medical Systems, Netherlands) and respiratory belt, respectively.

### MRI Data Acquisition

All MRI data were acquired on a 3-T Philips Achieva MRI scanner (Philips Medical Systems, Best, Netherlands) using a whole-body transmit coil and 32 channel head receive coil. Figure 1A shows the times that the different MRI sequences used were acquired relative to the gas challenges.

A multi-inversion time flow-sensitive alternating inversion recovery (FAIR) pulsed ASL sequence with two-dimensional echo-planar imaging (2D-EPI) readout (Kim, 1995) was used to acquire quantifiable measures of baseline perfusion and transit time. Imaging parameters used were: echo time (TE): 9 ms; repetition time (TR): 8 s; inversion times (TIs): 0.4, 0.6, 0.8, 1.0, 1.2, 1.4, 1.6, 1.8 s voxel size: 3.25 mm × 3.25 mm in plane; slice thickness: 5 mm; slices: 12. Four volumes of data were acquired for TIs between 0.4 and 1.4 s whilst 10 volumes of data acquired for TIs between 1.6 and 1.8 s, due to lower SNR at longer TIs. A base equilibrium M_0_ scan was acquired with the same parameters but without the inversion pulses required for ASL sequence (further details can be found in Burley, 2018; Burley et al., in press).

To allow measurement of CVR using MRI, a FAIR double acquisition background suppression (DABS) sequence (Mullinger et al., 2013, 2017) was used for simultaneous acquisition of ASL and BOLD data during the gas challenge protocol (Figure 1A). Imaging parameters used were: TR = 5.2 s (label-control pair), with background suppression pulses at T_*BGS1*_/T_*BGS2*_: 339 ms/899 ms, label delay = 1400 ms, TE = 9 ms (ASL)/40 ms (BOLD), voxel size: 3.25 mm × 3.25 mm × 5 mm, 12 slices of ASL and BOLD data, FOV: 212 mm × 212 mm, SENSE factor: 2.3, volumes: 205 (label-control pairs). A whole-head T1-weighted anatomical (magnetisation prepared rapid gradient echo; MPRAGE) image with 1 mm^3^ resolution was also acquired.

### TCD Data Acquisition

Blood velocity in the right and left MCA were measured simultaneously using TCD (Doppler Box, DWL, Compumedics Ltd, Germany), with a 2-MHz probe placed over each temporal window on the right and left side of the head. Probes were prepared with ultrasound gel and held in place with a headset. Search and identification procedures were done in accordance with established guidelines (Willie et al., 2011). Data were acquired from the whole of the gas challenge protocol from which both resting blood velocity and CVR metrics could be obtained, including a period of steady state (see Figure 1A).

## Study 2

### Participants

Twenty healthy volunteers in two age groups participated: 10 younger fit participants (22 ± 2 years) and 10 older participants (72 ± 4 years) self-reporting as “unfit.” Participants were excluded if they had any neurological or psychiatric conditions. Participants were not taking any medication (with exception of oral contraception for females) and had no history of cardiovascular, cerebrovascular or respiratory disease.

Fitness status for the younger group was determined by performance on a maximum oxygen consumption (V̇O_2_max) fitness test as described in Study 1 (>45 mL min^–1^ kg^–1^ placed them in the fit group). For the older group, the physical activity status was determined using a questionnaire (New Zealand Physical Activity and Readiness Questionnaire Short Form).

### Overview

Younger participants for this study completed six visits in total. The first visit included general health screening, MRI safety screening, fitness questionnaires. After inclusion into the study, the second visit included the aerobic fitness test on a treadmill or stationary bike to determine maximal oxygen consumption (V̇O_2_max) (see Supplementary Methods). The third visit was a familiarization of the CBF measures using TCD and gas challenges (not reported). After satisfactory completion of familiarisation trials (i.e., no adverse reactions to breathing the CO_2_ stimulus were experienced by the participant and adequate Doppler signals were identified), participants were invited back for the three full testing sessions. The final three visits involved collecting CBF and CVR measures, two using TCD and the other using MRI. The order of these three visits was randomised and counterbalanced between participants using a computer random number generator. The two visits using TCD were done to assess test-retest reliability of TCD measures, with the same researcher collecting all data. The older participants for this study completed four visits in total, they did not do the fitness test or the second TCD visit. As for Study 1, for all visits, participants were asked to avoid vigorous exercise and alcohol for 24 h, caffeine for 12 h and heavy meals for 4 h prior to study participation.

### Cerebrovascular Reactivity Test

Participants were asked to lie supine, relax and breathe as naturally as possible. The CVR test was performed after a resting period of at least 20 min. It consisted of two repeats of a 4-min period of breathing room air followed by 4 min of breathing a 5% CO_2_ stimulus, followed by a final 2 min of room air as shown in Figure 1B. The equipment used for gas delivery and heart rate monitoring was identical to Study 1, with the exception of the gas analyser for the MRI sessions. For this session, a dedicated CO_2_ analyser (CD-3A, AEI Technologies, United States) was used, which was calibrated the same way as the analyser used for TCD (using the same procedures as Study 1).

### MRI Data Acquisition

MRI data were acquired using the same hardware as in Study 1. Figure 1B shows the times that the different MRI sequences were acquired relative to the gas challenges.

A multi-inversion time FAIR pulsed ASL sequence with 2D-EPI readout, similar to Study 1, was used to acquire quantifiable measures of baseline perfusion and transit time. Imaging parameters used were: echo time (TE): 9.6 ms; repetition time (TR): 3 s; inversion times (TIs): 0.4, 0.6, 0.8, 1.0, 1.2, 1.4, 1.6, 1.8, 2.0 s voxel size: 3 mm × 3 mm × 5 mm; slices: 12. Four volumes of data were acquired for TIs between 0.4 and 1.4 s whilst 10 volumes of data acquired for TIs between 1.6 and 2.0 s. As for Study 1, base equilibrium M_0_ scan was also acquired. In addition, a whole head EPI with the same resolution and orientation as the ASL sequence to aid corregistration was acquired.

Blood flow in each vessel was measured using a VCG-gated 2D phase contrast angiography (PCA) on a single slice perpendicular to each targeted vessel of interest. Measures in the right and left MCA were acquired both during the baseline and gas periods (see Figure 1B). This allowed these PCA measures to determine blood velocity and flow during baseline (rest) and PCA-derived measures of CVR, akin to that acquired from TCD but using velocity and flow measures. Imaging parameters were: Cardiac cycle phases = 30, TR = 15 ms; TE = 3 ms, 10° flip angle, voxel size = 1.21 mm × 1.21 mm × 6 mm, Velocity encoding = 150 cm/s (allowing for increases during gas challenge), SENSE factor 2. The order of the PCA acquisitions was always right MCA followed by left MCA both during the baseline and gas challenge periods. During the gas challenge period the acquisition started 90 s after the 5% CO_2_ stimulus was started (see Figure 1B). The nominal time for the PCA acquisition was nominally 44 s, however, the time taken to acquire the PCA measures varied depending on how robust their VCG trace was as the R-peak needed to be detected.

To allow measurement of CVR from BOLD MRI in this study, a double-echo 2D-EPI sequence was used to acquire BOLD weighted fMRI data during the gas challenge as shown in Figure 1B (since the ASL data in Study 1 could not be used due to insufficient SNR [see analysis]). Imaging parameters were 3 s TR; TE_1_/TE_2_ = 20/45 ms, 3 mm^3^ voxel size, 38 slices, 345 volumes, SENSE factor 2. A whole head T1-weighted anatomical (MPRAGE) image with 1 mm^3^ resolution was also acquired.

### TCD Data Acquisition

TCD data were acquired using the same hardware and researcher as in Study 1. Again, data were acquired from the whole of the gas challenge protocol from which both resting blood velocity and CVR metrics could be obtained, see Figure 1B. Since 2 repeats of the stimulus were given this allowed two measures of baseline MCAv and the CVR to be determined without the TCD probe being moved. For the younger participants they returned for a second measurement session with the same set-up to test for repeatability of MCAv measures of resting blood velocity and CVR metrics.

## Analysis

For both TCD and MRI visits in both studies, collected gas fractions were converted to partial pressure measures using the barometric pressure of that day ((percentage CO_2_/100)^∗^(760-barometric pressure)).

## Study 1

The 7% CO2 was not tolerated by 4 of the participants, therefore CVR analysis was only performed on the 5% CO_2_ challenge.

### MRI

#### Calculating Resting Perfusion and Transit Times From ASL Data

Data were preprocessed using conventional methods in FSL^1^ (see Burley, 2018; Burley et al., in press for details). Resting cerebral perfusion (mL⋅100g^–1^⋅min^–1^) and transit time (seconds) measures were calculated using the FSL Bayesian Inference for Arterial Spin Labelling MRI (BASIL)^2^ toolset (Chappell et al., 2009). ASL data acquired at multiple TIs were fitted to the kinetic curve model (Lu et al., 2004) so that perfusion estimation errors associated with variable transit times across groups could be avoided.

Perfusion and transit times were assessed in all grey matter tissue as well as several regions of interest (RoIs) within the grey matter. RoI masks were defined from the conjunction of the relevant regions from the Harvard atlas (in FSL) and the normalised individual participant’s grey matter mask. RoIs used were: whole of right hemisphere, cingulate gyrus, frontal lobe, motor lobe, occipital lobe and parietal lobe, as previously employed (Thomas et al., 2013), and we detail elsewhere (Burley, 2018; Burley et al., in press). Mean cerebral perfusion and transit times across the participant groups were determined for the whole of the imaged grey matter and the different RoIs.

#### Calculating CVR From DABS Data

##### Pre-processing

The DABS data were separated into the BOLD and ASL data for subsequent analysis. The BOLD data were physiologically corrected for cardiac and respiratory effects using RETROICOR (Glover et al., 2000). All data were then motion corrected using FLIRT (Jenkinson et al., 2002) in the FMRIB Software Library (FSL, see text footnote 1). Using in-house Matlab programmes, separately the ASL data (acquired at TE = 9 ms) and BOLD data (acquired at TE = 40 ms) were linearly interpolated (Interp function, Matlab, Mathworks United States) to an effective TR of 2.6 s. Label-control pairs of ASL data were subtracted (using simple subtraction) to create perfusion weighted (CBF) images. BOLD-weighted image pairs were averaged to produce mean BOLD-weighted data. Further pre-processing was carried out in FSL. BOLD data were normalised to the standard Montreal Neurological Institute (MNI) template. After inspection of CBF data it was decided that further analysis could not be performed due to the inherently low SNR producing spurious variability during baseline and CVR gas challenges. Therefore, the post-processing stages outlined below were only carried out on the BOLD data to give a measure of CVR.

##### Post-processing

Firstly, the mean BOLD signal for each volume over the individual-subject grey matter tissue mask (i.e., excluding veins) was calculated. The vein mask was created by plotting a histogram of the percentage BOLD signal change to the entire gas challenge across the grey matter and where this change was greater than 15%, signal was regarded as a vein response and excluded. P_*ET*_CO_2_ readings were downsampled to the sampling rate of the BOLD data (2.6 s). To account for the delay in the P_*ET*_CO_2_ readings relative to the BOLD readings (due to the gas sampling line length), the mean BOLD signal intensity from the grey matter only was temporally aligned with the P_*ET*_CO_2_ (mm Hg) trace using an iterative process to maximise the correlation of the BOLD and P_*ET*_CO_2_ trace over the whole timecourse, as performed previously (Yezhuvath et al., 2009; Thomas et al., 2013).

A general linear model (GLM) was constructed containing eight regressors (P_*ET*_CO_2_ trace aligned to the BOLD signal, linear drift and six movement parameters) in SPM8^3^. To allow for the fact not all participants completed the 7% CO_2_ stimulus, the GLM only consisted of the data for the first 11 min of the DABS acquisition to only include the 5% CO_2_ challenge and surrounding baseline periods (see Figure 1A). The beta weight related to the P_*ET*_CO_2_ regressor at each voxel provided a metric of CVR in units of % BOLD signal change/mmHg P_*ET*_CO_2_ change at each voxel (Thomas et al., 2013). The mean GLM-derived CVR over all grey matter and different RoIs were calculated as was done for baseline CBF measures.

#### Calculating Baseline Blood Velocity and CVR Using TCD

Relative CVR was calculated using Equation 1.

(1) $$\mathrm{Relative}\,\mathrm{CVR}=\frac{100\times \left(\frac{V_{5\%\,{\mathrm{CO}}_{2}}-V_{\mathrm{base}}}{V_{\mathrm{base}}}\right)}{\mathrm{PETCO}\,2_{5\%\,{\mathrm{CO}}_{2}}-\mathrm{PETCO}\,2_{\mathrm{base}}}$$

where ***V****_*base*_* is the baseline (resting) velocity averaged over 60 s of stable resting data and averaged over the left and right hemisphere MCA. *V_5%CO2_* is the blood velocity during the 5% CO_2_ measured from 2.5 min into the stimulus duration and averaged over 30 s (see Figure 1A), in line with best practice (Burley et al., 2020), and averaged over the left and right hemisphere MCA. *PETCO2_base_* and *PETCO2_5%CO2_* are the mean P_*ET*_CO_2_ (mm Hg) signal from the same time windows as the blood velocity measures; for the baseline and 5% CO_2_ measures, respectively. The ***V****_*base*_* measure averaged over the right and left MCA was taken as the baseline velocity measure from the TCD acquisition.

It is important to note that MCA measures of blood velocity across hemispheres were averaged wherever possible as no significant difference (paired t-test) was found between hemispheres during baseline or gas challenge. However, in participants where only one MCA could be insonated, then this single recording was taken for that participant as no difference in MCAv is expected between hemispheres (Billinger et al., 2017; Lee et al., 2020). In a post-hoc analysis just the right MCA was used, with the same analysis performed on this single MCA, as analysis of Study 2 showed greater repeatability in right hemisphere TCD measures (see Results).

### Statistical Analysis

Change in P_*ET*_CO_2_ values from baseline to 5%CO_2_ were compared between TCD and MRI visits using a paired t-test to ensure they were not significantly different. Resting CBF and CVR measures were compared between age and fitness groups using 2-way ANOVA. We also separately compared younger fit and older unfit groups for both MRI and TCD outcomes using independent t-tests, decided *a priori* as the two groups with the potential to show the greatest separation (i.e., both age and fitness effects). The effect sizes were also calculated for between-group comparisons using Hedges’ g. Hedges’ g was used to calculate effect sizes between groups as the number of participants in each group were not the same. Correlational analyses were performed to investigate correlation between corresponding measures obtained with the two different imaging modalities.

## Study 2

### MRI

#### Calculating Baseline CBF Metrics From ASL and PCA Data

The analysis process for calculating perfusion and transit times from the ASL data in this study was identical to that outlined for Study 1.

PCA data were analysed using Q-Flow software (Philips Medical Systems). For the right and left MCA, a region of interest was drawn manually around the vessel lumen on each phase contrast image, with contour detection used. The mean signal intensity within each of the MCAs reflects blood velocity in that vessel (cm/s) for each cardiac phase, and the mean velocity across the cardiac cycle was computed. The cross-sectional area of each vessel lumen was multiplied by the mean velocity, to compute mean blood flow (ml/s) in each vessel. Measures from the right and left MCA were then averaged together as no significant difference (paired t-test) was found between hemispheres during baseline or gas challenge.

### Calculating CVR From BOLD Data

The double-echo EPI data were separated into the two echoes for subsequent analysis. The following analysis for each echo was as similar as possible to Study 1. For each echo, the BOLD data were physiologically corrected for cardiac and respiratory effects using RETROICOR (Glover et al., 2000). Data were then motion corrected using FLIRT (Jenkinson et al., 2002) in the FMRIB Software Library (FSL, see text footnote 1) and normalised to the standard MNI template.

First, the mean BOLD signal from the second echo for each volume over the individual-subject grey matter tissue mask (excluding veins) was calculated. P_*ET*_CO_2_ readings were downsampled to the sampling rate of the BOLD data (3 s). To account for the delay in the P_*ET*_CO_2_ readings relative to the BOLD readings (due to the sample line length), the P_*ET*_CO_2_ readings were temporally aligned to mean BOLD signal intensity from the grey matter as described in Study 1. For each echo, a general linear model (GLM) was constructed containing eight regressors (P_*ET*_CO_2_ trace aligned to the BOLD signal, linear drift and six movement parameters) in SPM8 (see text footnote 3). For each echo, the mean GLM-derived CVR measures (beta-weights) over all grey matter and different RoIs were calculated.

### Calculating CVR From PCA Data

Blood velocity and flow measures from the left and right MCA during the 5% CO_2_ gas challenge were obtained from the PCA data using the same analysis pipeline as outlined for the baseline PCA measures in this study. These values were average over right and left hemispheres. In addition, the mean P_*ET*_CO_2_ in the time windows corresponding to the periods when the baseline and gas challenge PCA data were acquired was calculated. Equation 1 was now used to calculate the relative CVR when using the blood velocity measured by PCA to input as ***V****_*base*_* and *V_5%CO2_*, and then using the blood flow (which takes account of changes in vessel diameter) to input as***V****_*base*_* and *V_5%CO2_*.

In addition, the same CVR metrics were determined for the right MCA only, as the reliability of TCD measures in the right hemisphere was found to be greater (see section “Results”).

### TCD

As for Study 1, and shown in Figure 1B, resting MCAv measures from the TCD data were taken from a 1-min average of stable resting data in the initial 5-min baseline. The blood velocity during the 5% CO_2_ stimulus was measured from 2.5 min into the stimulus duration and averaged over 30 s (see Figure 1B). CVR was then calculated using Equation 1.

The reliability of TCD measures was determined, since the accuracy of Doppler measures are known to be dependent on the operator and their experience (Willie et al., 2011; Thomas et al., 2015). The coefficient of variation (CoV [%]) (Bax et al., 2005) and the correlation between measures was then determined for each pair of measures within a visit (within visit reliability) and also between visits (younger group only, with second TCD visit) for measure 1 and 2 separately (between visit reliability). These measures were then averaged within and between participants to give a mean CoV and correlation of the TCD measures within and between visits for each group.

### Statistical Analysis

Change in P_*ET*_CO_2_ values from baseline to 5%CO_2_ were compared between TCD and PCA MRI visits using a paired *t*-test to ensure they were not significantly different. Group means of resting CBF and CVR measures were compared between the younger fit and older unfit groups using independent t-tests for BOLD, TCD and PCA results. The effect sizes were also calculated for between-group comparisons using Hedges’ g. Hedges’ g was used to calculate effect sizes between groups as the number of participants in each group were not the same. Correlational analyses were performed to investigate correlation of corresponding measures obtained across modalities.

### Pooling Study 1 and 2

To increase power and establish if trends seen separately in Studies 1 and 2 would result in overall patterns across all the data collected. BOLD and TCD CVR measures across the two studies were pooled. Since fitness status was not characterised in the same way for Studies 1 and 2 (with Study 1 likely to contain fitter older people in the unfit group than Study 2 as they had to have a clean ECG trace), data were only pooled and then grouped according to age. Over these pooled data, group means of CVR measures were compared between the younger and older groups using t-tests for BOLD and TCD results, and Hedges’ g used to calculate effects sizes for the between-group comparisons. Correlational analyses were performed to investigate correlation of measures obtained across modalities.

## Results

### Study 1

All data for BOLD and TCD CVR measures to be calculated were successfully acquired on 18 younger participants (25 ± 7 years, 7 female) and 12 older participants (69 ± 4 years, 5 female). Comparison of resting CBF measures between MRI and Doppler are reported elsewhere (Burley, 2018; Burley et al., in press). In brief, these showed a main effect of age with significant (*p* < 0.05) differences between younger and older groups in transit time (seconds) [*Hedges’ g* = 1.70], measured from ASL MRI, and MCAv (cm⋅s^–1^) [*Hedges’ g* = 0.95], as measured with TCD. The two imaging metrics also significantly negatively correlated with one another across the whole group (*r* = −0.63, *p* < 0.0001).

**FIGURE 2:**
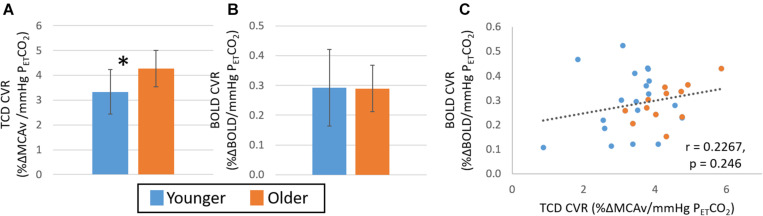
Study 1: Comparison of CVR measures with TCD **(A)** and BOLD **(B)** across younger (blue) and older (orange) groups. Error bars show the standard deviation across the group. * denotes a significant difference (*p* < 0.05, *t*-test) between groups. Panel **(C)** shows the correlation of CVR measures from BOLD and TCD data, no significant correlation over the whole cohort (*n* = 30) is seen. TCD, transcranial Doppler; BOLD, blood oxygen level dependant.

There were no significant main effects for fitness in any of the measures of CVR or within either age group (all *p* > 0.05). As shown in Figure 2, there were differences between younger and older groups for CVR measures, however, these were dependent on imaging modality. Specifically, while a significantly greater CVR response was observed in the older than the younger group for the TCD-CVR measure (*p* < 0.05, *Hedges’ g* = 1.13), there was no significant difference between age groups over the whole grey matter for the BOLD-CVR measure (*p* = 0.104, *Hedges’ g* = 0.11). Furthermore, no significant differences were seen in any of the sub regions assessed (see Supplementary Figure 1). Given this disparity between TCD and BOLD group measures, it is not surprising that no significant correlation was seen between the BOLD and TCD CVR measures (Figure 2C; *r* = 0.23, *p* > 0.05).

**FIGURE 3:**
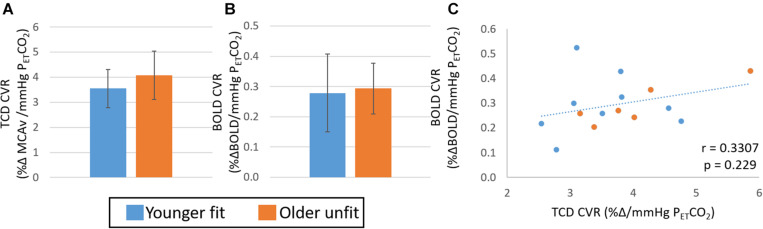
Study 1: Comparison of CVR measures with TCD **(A)** and BOLD **(B)** across younger fit (blue) and older unfit (orange) groups. Error bars show the standard deviation across the group. No significant differences (*p* > 0.05, *t*-test) between groups are seen. Panel **(C)** shows the correlation of CVR measures from BOLD and TCD data, no significant correlation over the whole cohort (*n* = 15) is seen.

Extreme group comparisons of the two groups with the expected most likely difference, younger fit (group size: 9, 28 ± 8 years, 2 female) and older unfit (group size: 6, 70 ± 5 years, 4 female), revealed a similar pattern in the data; although there was no significant difference between the CVR measures obtained from either TCD (Figure 3A, *Hedges’ g* = 0.63) or BOLD (Figure 3B, *Hedges’ g* = 0.03) data. In addition, no significant relationship between BOLD and TCD CVR measures was seen across this sub-group of the data (Figure 3C; *r* = 0.33, *p* > 0.05).

### Study 2

TCD and BOLD data required to calculate CVR measures were successfully acquired on 10 younger fit participants (22 ± 2 years, 3 female V̇O_2_max = 52 ± 7 mL⋅kg^–1^⋅min^–1^) and 8 older unfit participants (72 ± 4 years, 2 female). Two of the older participants did not manage to complete the whole MRI visit. In addition, resting ASL data were successfully acquired on all of these 18 participants, as well as PCA data both at rest and during 5% CO_2_, although one PCA data set for an older participant was missed due to technical problems.

Consistent with findings from Study 1, measures of resting CBF from TCD MCAv and ASL MRI transit time were significantly negatively correlated (*r* = −0.61, *p* < 0.05) across the whole sample when considering whole grey matter and average MCAv across hemispheres (see Supplementary Figure 2A). There was a complementary relationship between the whole grey matter transit times and the average MCA velocity (Supplementary Figure 2B, *r* = −0.68, *p* < 0.05) and flow (Supplementary Figure 2C, *r* = −0.68, *p* < 0.05) measures derived from the PCA MRI data. In addition, a significant difference (*p* < 0.05) between younger and older groups was seen for these baseline CBF measures in all modalities (Supplementary Figure 3). In the younger group, resting CBF had significantly higher velocity (measured with both TCD and PCA) and flow (measured with PCA), which was reflected by shorter transit times (measured with ASL). However, while the mean value was lower in the older group, there was no significant difference in baseline perfusion (measured with ASL) in the grey matter between groups (Supplementary Figure 3D).

**FIGURE 4:**
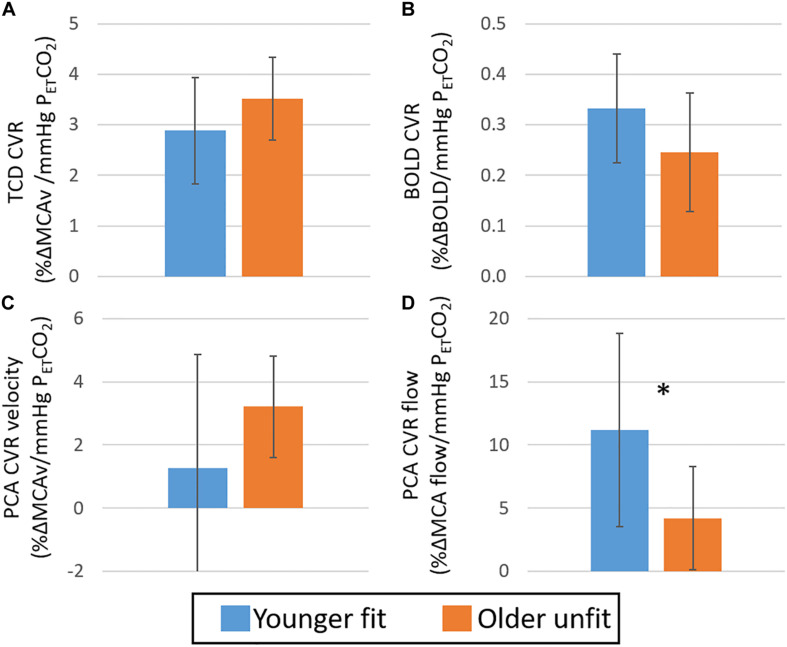
Study 2*:* Comparison of CVR derived from TCD MCAv **(A)**, BOLD **(B)**, PCA MCA velocity **(C)** and PCA MCA flow **(D)** across younger fit (blue) and older unfit (orange) groups. Error bars show the standard deviation across the group. * denotes a significant difference (*p* < 0.05, *t*-test) between groups.

**FIGURE 5:**
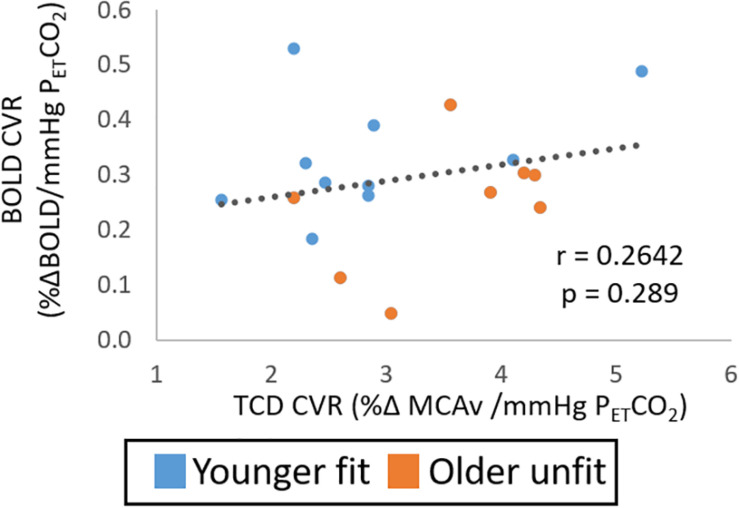
Study 2*:* Shows the relationship of CVR measures from BOLD and TCD data. No significant correlation over the whole cohort (*n* = 18) is seen.

When considering differences in CVR between these targeted groups of likely difference, no significant differences were seen between the two groups for the TCD- or BOLD-CVR measures (Figure 4A; *p* > 0.05, *Hedges’ g* = 0.65 and B; *p* > 0.05, *Hedges’ g* = 0.70, respectively); although some sub regions interrogated with the BOLD-CVR measures did show significantly lower BOLD CVR in the older unfit group (Supplementary Figure 4). Further, no difference was seen in the CVR derived from PCA-MCA velocity (Figure 4C *p* > 0.05, *Hedges’ g* = 0.67). However, a difference was seen when MCA flow, which accounts for changes in vessel diameter and blood velocity, was used to derive CVR (Figure 4D *p* < 0.05, *Hedges’ g* = 1.08). Although not significant, the pattern of difference in CVR derived from both MCA velocity measures (TCD and PCA-MRI) is that the younger group have a lower CVR (Figures 4A,C) than the older group, also in agreement with Study 1 (Figures 2A, 3A). However, the CVR derived from the BOLD and PCA-MCA flow data show the younger group have a higher CVR (Figures 4B,D and Supplementary Figure 4) than the older group. Consistent with these disparate observations between modalities, there was no correlation between CVR measures derived from the TCD and BOLD data (see Figure 5; *r* = 0.26, *p* > 0.05). In addition, despite the similar pattern in MCA velocity-derived CVR measures from TCD and PCA, these measures of CVR also showed no significant correlation over the group (Supplementary Figure 5A; *r* = −0.19, *p* > 0.05). Similarly, no relationship between BOLD and PCA MCA flow was found (Supplementary Figure 5B; *r* = 0.04, *p* > 0.05).

### Pooling Study 1 and 2

**FIGURE 6:**
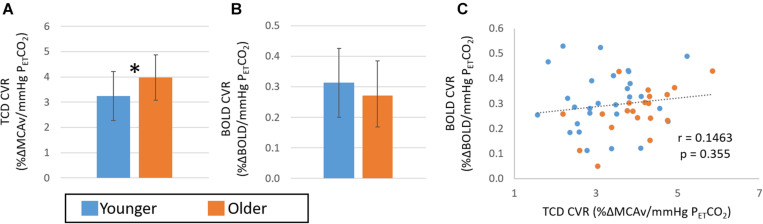
Comparison of CVR measures pooled over Study 1 and 2 with TCD **(A)** and BOLD **(B)** across younger (blue) and older (orange) groups. Error bars show the standard deviation across the group. * denotes a significant difference (*p* < 0.05, *t*-test) between groups. Panel **(C)** shows the correlation of CVR measures from BOLD and TCD data; no significant correlation over the whole cohort (*n* = 48) is seen.

The data pooled across Studies 1 and 2 allowed investigation of age effects in a larger group. This resulted in 28 participants (24 ± 6 years, 10 female) in the younger group and 20 participants (57 ± 5 years, 7 female) in the older group. Figure 6 shows the group differences in CVR determined from TCD (Figure 6A) and BOLD (Figure 6B). These pooled data revealed significantly (*p* < 0.05, *Hedges’ g* = 0.75) greater CVR in the older than the younger group as determined from TCD measures. Further, whilst no significant difference was seen between groups in the CVR from the BOLD measures over the whole head (*p* > 0.1, *Hedges’ g* = 0.38), there was a trend for a smaller CVR in the older group than the younger group in the cingulate (*p* = 0.06; *Hedges’ g* = 0.53), and motor (*p* = 0.05; *Hedges’ g* = 0.61) regions. Consistent with these opposing findings, there was no correlation in CVR determined from TCD and BOLD across the whole group (Figure 6C; *r* = 0.15, *p* > 0.05).

### Source of CVR Discrepancy

Given the opposing CVR findings between TCD- and BOLD-derived measures, we explored potential sources of the discrepancy that included any potential source due to the reliability of the TCD measure. It has been suggested BOLD-CVR should be acquired at a shorter echo time (Liu et al., 2019), therefore the relationship of CVR between echo 1 and 2 of these BOLD data were investigated. Study 2 showed the CVR calculated from both BOLD echo times correlated extremely well (Supplementary Figure 6A; *r* = 0.94, *p* < 0.05). Furthermore, the CVR derived from BOLD echo 1 data and CVR derived from TCD data were not correlated (Supplementary Figure 6B; *r* = 0.20, *p* > 0.05), reflecting the same finding shown in Figure 5 for BOLD echo 2 data.

The test-retest reliability data of baseline TCD measures acquired on the participants in Study 2 showed that TCD results were of a high standard with low coefficients of variation and a high degree of correlation between measures taken on the same day and between days (Table 1). There was a slightly greater variation (lower correlation, higher CoV) between MCAv measures during the CO_2_ challenge than during the resting baseline periods, particularly between visits. Although the reliability of TCD measures of MCAv during rest or gas challenge was good, the reliability of the CVR measures within or between visits was found to be consistently lower (higher CoV and lower correlation). Across the different measures and comparisons the reliability appeared to be poorer in the left MCAv measures. Therefore, it is possible noise in the left MCAv measures were driving the lack of correlation between TCD- and BOLD-derived CVR measures. Given these findings, all of the CVR data were re-calculated using only the right hemisphere of the brain, that is the right MCA (for TCD and PCA measures) and the grey matter from the right hemisphere (for BOLD measures). However, this did not change any of the findings for the relationship between CVR derived from different modalities in Study 2 (Supplementary Figure 7). When considering the combined data from Studies 1 and 2 and only the right hemisphere, there was no significant correlation (*r* = 0.26, *p* > 0.05) between the TCD- and BOLD-derived CVR measures observed (Supplementary Figure 8C) nor was there an agreement between the TCD and BOLD CVR measures for younger and older groups (Supplementary Figures 8A,B).

**TABLE 1:** Study 2: Results of test-retest reliability assessment of the TCD measures. Assessment of the coefficient of variation (CoV) and correlation between measures collected on the right and left MCA during baseline (resting) period and during 5% CO_2_ gas challenge.

		**Baseline**		**CO_2_ gas challenge**		**CVR**		
		**Right MCA**	**Left MCA**	**Right MCA**	**Left MCA**	**Right MCA**	**Left MCA**	**Average MCA**
Younger: mean within visit measures	CoV (%)	3.8	3.6	2.9	2.7	27	22	28
	Correlation (r)	0.97	0.92	0.98	0.98	0.66	0.59	0.56
Older: mean within visit measures	CoV (%)	4.0	4.2	2.9	1.7	12	14	15
	Correlation (r)	0.98	0.99	0.98	0.96	0.68	0.45	0.46
Younger: mean Between visit measures	CoV (%)	6.4	9.4	7.8	8.8	33	29	29
	Correlation (r)	0.92	0.75	0.89	0.83	0.33	0.34	0.26

*The reliability of the calculated CVR value is also presented. For younger people measures are averaged over visits/measures one and two as applicable.*

## Discussion

CVR metrics derived from both TCD and BOLD-MRI measures are reported to be a biomarker of brain health (e.g., Kleiser and Widder, 1992; Sur et al., 2020), and even shown to have prognostic value for stroke risk (Silvestrini et al., 2000; Portegies et al., 2014; Smeeing et al., 2016). Given this, one might assume that between-group differences across modalities would be the same. However, as highlighted in the introduction, there are many examples in the literature of within and between imaging modality inconsistencies and contradictions for this brain health metric, which may originate from what is measured by the different imaging approaches. Here, we sought to quantify expected age and fitness differences in the CVR outcome measure between these two commonly used imaging modality approaches. We also assessed other MRI metrics of CBF in order to compare how more direct measures of CBF align with TCD-derived CVR, and resting measures between our groups.

Overall, we found complementary relationships between baseline CBF measures across our cohort for all modalities (TCD MCAv, PCA MCA velocity and flow, and perfusion transit time) (Supplementary Figures 2, 3; also see Burley, 2018; Burley et al., in press) and a significant difference between younger and older groups, with the older group showing lower blood velocity and flow in arteries, and longer time for blood to reach the capillaries. These clear group differences in baseline measures are in line with previous reports (TCD: Ainslie et al., 2008; Bailey et al., 2013; MRI: Liu et al., 2012). However, the relationship between the CVR metrics derived from the two imaging modalities was less clear. Most importantly, we observed no within-participant correlation between the CVR metrics calculated from TCD and BOLD MRI over whole datasets (Figures 2C, 5, 6C). When considering differences between groups, we found that CVR was significantly lower in the younger group than the older group when derived from the TCD MCAv data (Figures 2A, 4A, 6A); whereas a trend in the opposite direction was seen with BOLD MRI, with higher CVR for the younger group than the older group (Figures 2B, 4B, 6B). It is worth noting that the direction of this BOLD-CVR trend is in better agreement with most of the age-related CVR changes reported in the literature (Bakker et al., 2004; Lu et al., 2011; Bailey et al., 2013; Oudegeest-Sander et al., 2014; Gauthier et al., 2015; Bhogal et al., 2016; Peng et al., 2018; Miller et al., 2019), which includes both MRI and TCD-derived approaches. Our findings may explain the discrepancies that are currently being reported in the literature when comparing fit and unfit groups or younger and older cohorts (as illustrated in the Introduction), where either BOLD MRI or TCD are being used to determine CVR. The following discussion will examine, based on our findings, the possible sources of the differences in CVR results across imaging modalities, and suggest what could be done to ensure that CVR is a reliable metric of brain health and biomarker in the future.

### Sources of CVR Differences Across Imaging Modalities

#### Measurement Inaccuracy and Analysis Methods

Perhaps the easiest explanation for the lack of CVR relationships would purely be insufficient data quality in either one of the imaging modalities to allow comparison across modalities. However, if this were the case we would expect no relationship to be seen in the baseline measures across imaging modalities, which is not the case. Indeed, a strong relationship between CBF measures from the different modalities of TCD, PCA MRI and ASL MRI was observed (Burley, 2018; Burley et al., in press; Supplementary Figures 2, 3) and the between-group differences obtained with different modalities agreed with one another in these resting CBF measures, which was not seen in the CVR measures.

The acquisition of the TCD measures is the most challenging of the measures taken in this study, as it requires the researcher to find the MCA in the same way each time (Willie et al., 2011); potentially making the reproducibility of these measures the lowest. Therefore, the test re-test reliability of the TCD data was assessed in Study 2 to determine if the CVR discrepancies observed in the conventional TCD and MRI measures could all be explained by poor repeatability. When comparing TCD data to MRI data the most relevant measure is the between visits measure, which still shows a low CoV and strong correlation between MCAv measures taken in different visits (Table 1). Furthermore, there is not a clear change in the reliability of this measure when the participant is breathing the gas compared with the baseline measures. Whilst a strong correlation was seen between the MCAv measures done on different days with TCD; the relationship between TCD CVR measures from different days was weaker (Table 1). However, the correlation between TCD CVR measures (average *r* = 0.43) taken was still much greater than the relationship of the TCD CVR measures to the CVR measures derived from PCA MRI (Supplementary Figure 5A, *r* = −0.19), which would have been expected to provide similar CVR measures. To fully be able to interpret these results it would be necessary to also assess test re-test reliability in the MRI CVR measures to try and disentangle if it is participant physiology (Shoemaker et al., 2020) or measurement technique that is driving this variability and is contributing to the lack of correlation between modalities. Nevertheless, the variability that we observed with the TCD-CVR is consistent with other studies that have reported CoV for this measure (Shoemaker et al., 2020) and/or examined its variability (Burley et al., 2020). Finally, the PCA MRI-derived flow measure of CVR in the MCA, which is not affected by the operator to the same extent as TCD acquisition, also did not correlate with the BOLD CVR metrics (Supplementary Figure 5B).

In addition, if random noise is the reason that a correlation is not seen between TCD and MRI CVR measures, then a larger cohort than that used in these studies would provide further insight into the relationship between measures. However, if this is the case then the utility of CVR as a biomarker (see later discussion) would become questionable, as when we combined our data across both studies we had measures on 48 people included (Figure 6C). Whilst a limitation of this work is the number of people who took part in Study 2, the additional information acquired on these participants was designed to inform the overall finding across all data. Indeed, this combined cohort from Studies 1 and 2 confirmed our initial observations that there was not a clear correlation between CVR metrics derived from TCD and BOLD measures.

An alternative explanation for the lack of correlation between CVR metrics is how CVR is calculated for different imaging modalities. It is known that the segment of data that is used to calculate CVR can affect the CVR outcome measure and therefore this might, at least in part, explain the lack of correlation (Burley et al., 2020). For BOLD data, the typical analysis strategy is to calculate CVR from the entire BOLD and P_*ET*_CO_2_ timecourse, via a GLM (Liu et al., 2019), which was employed as the primary method in the current work. By doing this the analysis includes the transition periods as the participant goes on and off the gas stimulus, and thus the integrated ventilatory response that influences this open circuit-type of CVR test (Battisti-Charbonney et al., 2011; Duffin et al., 2017) and the time course of this effect (Burley et al., 2020), which could affect the calculated CVR. Whereas, CVR determined from TCD data is calculated through a relative change of average blood velocity during the gas challenge, once a steady state is reached, from the average baseline blood velocity (Fierstra et al., 2013), thus not including the transition periods.

Given the differences in how the CVR metric is typically derived for different imaging modalities, we investigated if these differences may be driving the discrepancy in the CVR outcome measure across modalities. Using the data obtained in Study 1 we also checked to see if the analysis strategy explained the differences between CVR metrics obtained in different imaging modalities. We found that if the BOLD data signal was taken from the whole of the grey matter mask and then CVR was calculated using the same 30-s time windows used in the TCD analysis, there was no clear effect between groups on the CVR measure calculated. Similarly, if TCD data were used as a continuous time course (including the transition periods) and linearly regressed against the P_*ET*_CO_2_ measures, akin to a GLM, then again no clear effect on the CVR measures was seen. Thus, general patterns between groups remained the same when CVR was calculated in different ways from the BOLD data (see Figure 5.4, Burley, 2018), and, if anything, making the difference seen more pronounced and therefore a bigger discrepancy with CVR derived from TCD data. As such, the analysis methodology is unlikely to be the source of differences seen between our CVR measures derived from TCD and BOLD MRI.

### Biophysical Origins

The simplest explanation in terms of the vascular response that might be causing the differences in CVR is the fact that both TCD and BOLD MRI are providing indirect measures of CBF, which is the parameter of interest for characterising CVR. When considering CVR calculated from TCD, the changes in vessel diameter with CO_2_-induced vasodilation are not accounted for Ainslie and Hoiland (2014); Verbree et al. (2014); Coverdale et al. (2017). Thus, velocity increases may be greater, but diameter increases less in the older group with the gas challenge; causing opposing CVR changes between groups across measures. It is hard to predict the dominance of these two effects on the BOLD signal, which will reflect the accumulation of velocity and diameter changes over the cerebrovascular tree given BOLD reflects overall concentration of deoxyhaemoglobin within the blood. The general hypothesis that there are changes in velocity and vessel diameter that both alter with age is supported by the work of Coverdale and colleagues, who found that the percentage change of the MCA cross sectional area was smaller for older people than younger people when given a gas challenge (Coverdale et al., 2017). Indeed, our PCA MRI data also supports this hypothesis as it showed opposite effects between the younger and older groups when the MCA blood velocity (Figure 4C) or the MCA blood flow (Figure 4D) were used to determine CVR. However, our PCA measures of CVR did not significantly correlate with the TCD- or BOLD MRI-derived CVR measures over the whole group (Supplementary Figure 5). To verify this putative explanation for the differences between groups, data are required that accurately maps the blood vessel diameter changes with gas challenge compared with rest in different cohorts, building on the work of Verbree and colleagues (Verbree et al., 2014) and relating this to CVR measures obtained in the same session. It should also be noted with regards to cross modality comparison, that velocities measured with PCA are typically found to be 30–40% lower than those measured with TCD (Juttukonda and Donahue, 2019), however, as this is thought to be a systematic offset it is unlikely to explain our data. We suggest that whilst vessel diameter changes are likely to be some of the source of discrepancy, caution should be taken that this can fully explain the differences given our PCA MRI results.

It is known that the BOLD signal is affected by many factors other than changes in CBF. For example, changes in blood volume alone can cause changes in the BOLD signal, where an increased blood volume, with no changes in CBF or CMRO_2_, results in a decrease in the BOLD signal (Buxton et al., 1998). As discussed above, if vessel diameter changes with CO_2_ challenges are different between age groups, this could be contributing to the overall CVR metrics calculated from the BOLD signal. This is because it is well known that increases in cerebral blood volume driven by increases in vessel diameter reduce the BOLD signal (if there are no changes in CMRO_2_ or CBF), whereas, increases in CBF will increase the BOLD signal (Buxton et al., 1998). Therefore these two changes will have opposing effects on CVR determined by BOLD. Furthermore, baseline levels of haematocrit will also affect the amplitude of the BOLD signal change with the CO_2_ stimulus (Xu et al., 2018). As haemoglobin decreases with age (Mahlknecht and Kaiser, 2010), a smaller CVR response with increased age could be explained by these altered baseline levels of haemoglobin. Any change in haematocrit is not going to be reflected in the TCD or PCA-MRI data, which may explain why the CVR metrics calculated from both the TCD and PCA-MRI do not correlate with the BOLD-MRI CVR metrics.

An alternative, and most likely, explanation is that CVR metrics made with BOLD MRI and TCD are measuring different parts of the vascular tree. TCD is measuring blood velocity changes in the arteries, whilst BOLD MRI is primarily measuring the change in concentration of deoxy-haemoglobin in the venules and veins. Simplistically, one could assume that this difference may not matter as the blood going into the brain through the arteries must come out through the veins, and therefore changes in CBF must be reflected in both the arteries and the veins. However, the changes in vessel diameters due to CO_2_ are not uniform throughout the vascular tree (Piechnik et al., 2008). Moreover, if these changes in vasodilation across the vascular tree, for example driven by changes in endothelial function, are also dependent on the tested cohort (i.e., age or pathology) then CVR metrics calculated in different parts of the vascular tree will not correlate. Several studies have provided some evidence to support this explanation. For example, different CVR in large veins compared with large arteries has been shown using TCD (Valdueza et al., 1999), although these measures are clearly limited by unknown changes in vessel diameter. Using PCA-MRI, Geurts and colleagues have recently shown that there is a difference in CVR measured in arterioles compared with large arteries (Geurts et al., 2018). Indeed, other Doppler studies have shown that not even all arteries behave the same with regards to CVR (Sato et al., 2012; Willie et al., 2012; Carr et al., 2020). Therefore these studies suggest that there is a vessel type dependence for CVR measures (Schlader et al., 2013).

Taken together, the previous studies (Valdueza et al., 1999; Sato et al., 2012; Willie et al., 2012; Geurts et al., 2018; Carr et al., 2020) and our findings may indicate there are differences in the vascular reactivity of the arter(iole)s compared with the venules and veins, which change with age and perhaps also pathology. Different imaging techniques are sensitive to different parts of this vasculature tree. Therefore, across modality comparisons of CVR results should be performed with extreme caution. Unfortunately this has not been done within the literature to date, likely due to an expectation that these measures were reporting the same effect. However, with the work presented herein and the work of others there is an emerging picture highlighting that a combined imaging approach may allow us to differentiate more precisely which part of the vascular tree is compromised in a particular cohort, and thus providing a greater understanding of the pathology and treatment paths in the future. If proven through future work, this will be vital for the use of CVR as an accurate biomarker as is being proposed (e.g., Smeeing et al., 2016; Juttukonda and Donahue, 2019; Sur et al., 2020).

Future work to help further understand the results presented here should include simultaneous TCD and MRI measures. Whilst considerable effort was made in the current work to make the MRI and TCD session measurements as similar as possible (e.g., standardised exercise and diet, and matched time of day), there are still considerable differences in CVR measures taken on different days that are believed to have biophysiological origins (Shoemaker et al., 2020). Indeed, we have previously shown the TCD-based CVR measures can have considerable variation even when repeated on the same day within the same session (Burley et al., 2020). Therefore, we suggest that to eliminate the source of CVR discrepancy being due to different biophysiological responses on different days or times it would be necessary to make simultaneous recordings with the MRI and TCD approaches. In addition, future investigation to prove the hypothesis that different aspects of the vascular tree are reacting in different ways could include 4D flow PCA measures (Miller et al., 2019) at ultra-high field (Kang et al., 2016) to potentially allow the investigation of blood velocity and flow changes in arteries, arterioles, venules and veins simultaneously during gas challenges. However, considerable work will be required to optimise data acquisition to make such measures feasible as the baseline velocities across the vascular tree and changes in these with gas challenge would mean using a single velocity encoding value would limit the sensitivity of the technique to some of the vascular tree.

In conclusion, we have shown that despite clear relationships between baseline CBF measures from TCD and MRI and significant group differences (younger compared with older participants), there were no direct, within participant, correlations between CVR metrics calculated from TCD and MRI (BOLD and PCA) measures during a 5%CO_2_ challenge. Consistent with this lack of correlation, we showed opposing group effects between younger and older participants for CVR outcomes from TCD compared with BOLD MRI (i.e., CVR higher in older than younger for TCD, but vice versa for BOLD MRI). These findings challenge the validity of comparing CVR metrics across studies using different imaging modalities, but rather highlight that extreme caution should be taken if comparing CVR measures from a different imaging modality in another published study. However, since TCD and BOLD MRI target different parts of the vascular tree, they may be used together in the same cohorts to better understand the part(s) of the vascular tree that are compromised in a particular disease, providing a more detailed biomarker than either modality on its own. Nevertheless, further work is needed to better understand the source of imaging metric discrepancies in order that they may be better utilised for complementary, targeted, multimodal imaging to determine brain health.

## Data Availability Statement

The raw data supporting the conclusions of this article will be made available by the authors, without undue reservation.

## Ethics Statement

The studies involving human participants were reviewed and approved by The Science, Technology, Engineering and Mathematics Ethical Review Committee, University of Birmingham. The participants provided their written informed consent to participate in this study.

## Author Contributions

CB, SF, SL, and KM contributed to the conception or design of the work. CB, SF, KT, AW, SL, and KM contributed to the acquisition, analysis, or interpretation of data for the work, contributed to drafting of the work or revising it critically for important intellectual content. All authors approved the final version of the manuscript. All authors agreed to be accountable for all aspects of the work in ensuring that questions related to the accuracy or integrity of any part of the work are appropriately investigated and resolved. All persons designated as authors qualify for authorship, and all those who qualify for authorship are listed.

## Conflict of Interest

The authors declare that the research was conducted in the absence of any commercial or financial relationships that could be construed as a potential conflict of interest.

## Footnotes

1 https://fsl.fmrib.ox.ac.uk/fsl/fslwiki

2 https://fsl.fmrib.ox.ac.uk/fsl/fslwiki/BASIL

3 https://www.fil.ion.ucl.ac.uk/spm/
