# Supplementary material for: Contrasting Measures of Cerebrovascular Reactivity Between MRI and Doppler: A Cross-Sectional Study of Younger and Older Healthy Individuals
Source: Front Physiol. 2021 Apr 12;12:656746. doi: 10.3389/fphys.2021.656746 (PMC8072486; doi:10.3389/fphys.2021.656746)
Supplement: Supplementary file 1 [file Data_Sheet_1.PDF]

Supplementary Material

Methods

Study 1

Electrocardiogram and General Health Screening

Participants underwent a pre-exercise evaluation for general health screening. Participants >50 years completed a resting 12-lead ECG assessment and resting blood pressure measurement, which was reviewed by a cardiologist. Exclusion criteria included: family history of heart attack, high resting blood pressure (systolic >160, diastolic >90), ECG abnormalities (S-T suppression, >3 ectopic beats in a row, referral to GP advised).

Aerobic Fitness Assessment

Aerobic fitness was determined from a maximal oxygen consumption ($\dot{V}O_2$max) test. After screening and inclusion into the study, all participants completed a maximal aerobic fitness test to determine $\dot{V}O_2$max. Participants could choose to either cycle on an electromagnetically braked cycle ergometer or run on a treadmill. Although these approaches have been shown to yield different VO_2max values (Loftin et al. 2004), we considered the choice was justified so that older adults who were more likely to be frail or have other movement limitations (particularly those who were less fit) were more likely to be able to complete the test. The respiratory exchange ratio (RER), heart rate and rate of perceived exertion (RPE) were all monitored throughout to determine a valid fitness test (Riebe et al. 2018).

For the cycling protocol, participants were asked to cycle at a rate of ~70 rpm (rotations per minute) or above throughout, whilst the workload increased in increments of between 20 and 35 Watts (depending on age and fitness) every three minutes. For the running protocol, initial pace began at a speed where heart rate was approximately 65% of their predicted maximum, and then increased by 0.5 to 1.0 $km \cdot h^{-1}$ (depending on age and fitness) every two minutes for the first four stages, and then at a 1% incline per minute. For both protocols, the workload continued to increase until the participant reached volitional exhaustion or heart rate reached 100% of the participant's estimated maximum heart rate. Respiratory gases and gas volume were collected for measurement of the rate of VO_2. $\dot{V}O_2$max was then calculated from a 30-second average around the peak $\dot{V}O_2$ (i.e. highest value) and divided by body weight (kilograms).

References

Loftin M., Sothern, M., Warren, B., & Udall, J. (2004). Comparison of VO_2 peak during treadmill and cycle ergometry in severely overweight youth. Journal of Sports Science and Medicine, *3*(4), 554–560.

Riebe D, Ehrman JK, Liguori G, et al. ACSM's guidelines for exercise testing and prescription. 10th ed. Philadelphia: Wolters Kluwer 2018. https://www.academia.edu/36843773/ACSM_Guidelines_for_Exercise_Testing_and_Prescription_10th

Results

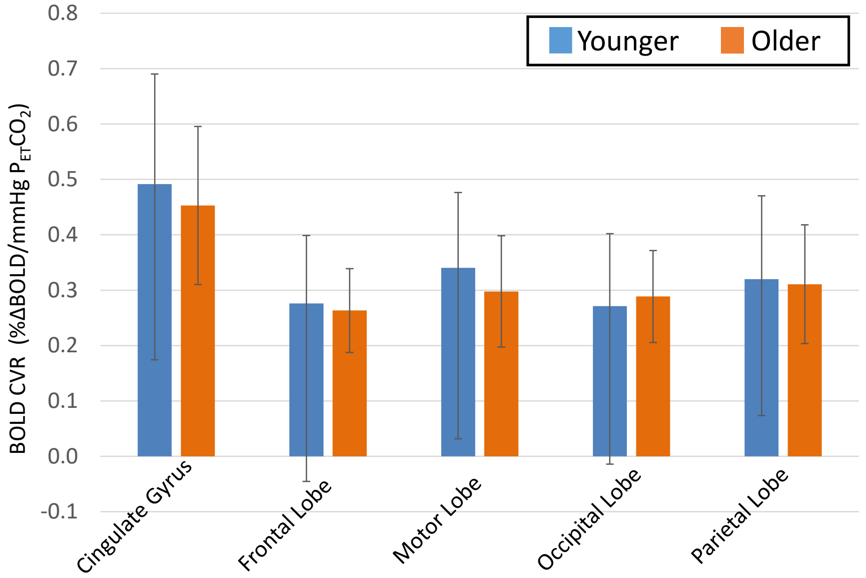

Figure S1: *Study 1:* Comparison of BOLD CVR measures across younger (blue) and older (orange) groups for different brain regions. Error bars show the standard deviation across the group. No significant differences between groups are seen. *BOLD* = blood oxygen level dependant; *CVR* = cerebrovascular reactivity

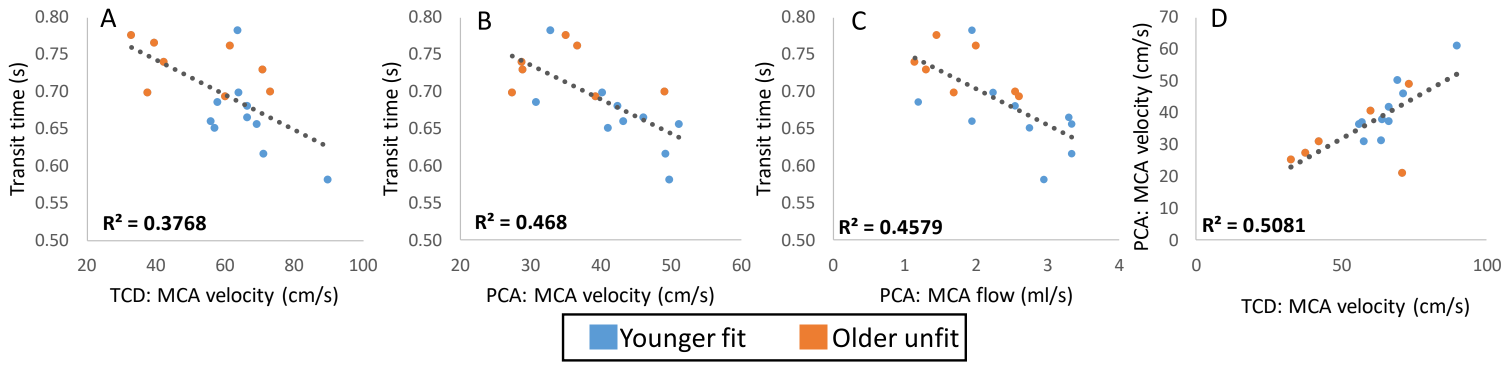

Figure S2: Relationship between baseline measures of CBF from TCD and MRI across the whole group in *Study 2*. **A**: Average MCAv measured with TCD is correlated with Transit time from MRI ASL. **B**: Shows the relationship of average MCAv measured with MRI PCA with Transit time from MRI ASL. **C**: Shows the relationship of average MCA flow measured with MRI PCA with Transit time from MRI ASL. **D**: Shows the relationship of average MCAv measured with TCD and MRI PCA. All four plots show a significant correlation over the whole cohort ($p<0.05$) of the various CBF measures in the MCA with the perfusion transit times of the grey matter tissue.

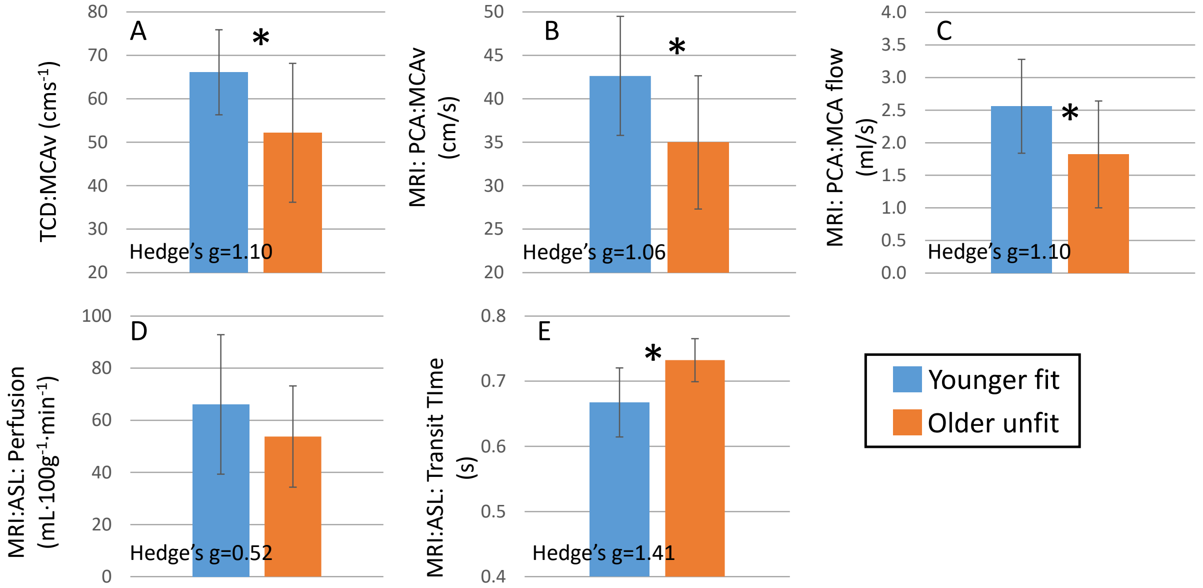

Figure S3: *Study 2:* Difference in baseline CBF measures across younger and older groups. **A**: MCAv measured with TCD, **B**: MCA velocity measured with MRI PCA, **C**: MCA flow measured with MRI PCA, **D**: Perfusion measured with MRI ASL, **E**: Transit time measured with MRI ASL. * denotes significant difference ($p<0.05$, t-test) between groups

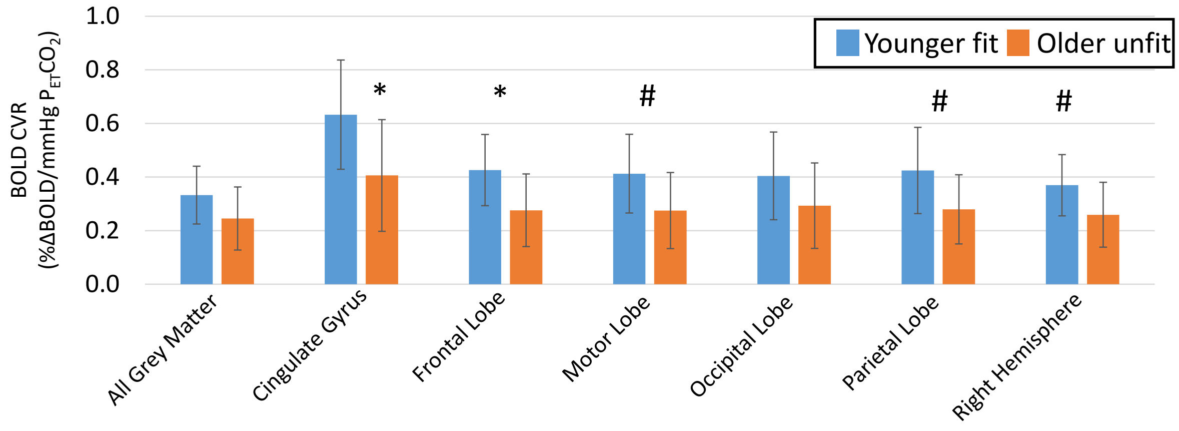

Figure S4: *Study 2:* Comparison of CVR measures derived from BOLD in different brain regions across younger fit (blue) and older unfit (orange) groups. Error bars show the standard deviation across the group for a region. * denotes a significant difference ($p<0.05$, t-test) between groups for that region, # denotes a trend ($p<0.1$, t-test) between groups for that region.

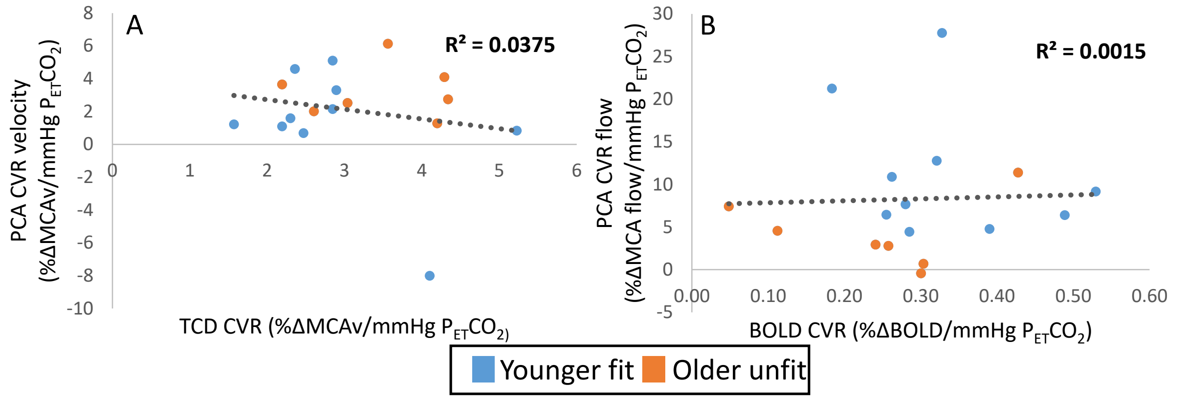

Figure S5: *Study 2:* Shows the correlation of CVR measures derived from: (**A**) TCD MCA velocity and PCA MCA velocity measures and (B) BOLD and PCA MCA flow measures. No significant correlations over the whole cohort were found.

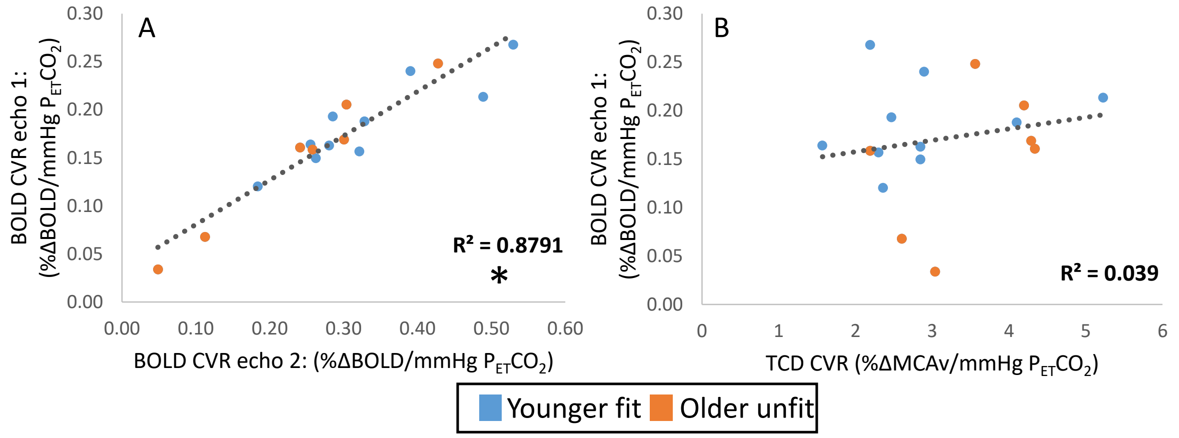

Figure S6: Data from Study 2. **A**: Shows the relationship of CVR measures from BOLD data acquired from echo 1 (TE = 20ms) and echo 2 (TE= 45ms). **B**: Shows the relationship of CVR measures from BOLD data acquired from echo 1 (TE = 20ms) and TCD data (compared with Fig 4). * denotes a significant correlation ($p<0.05$). Correlations were calculated over the whole cohort (young and old).

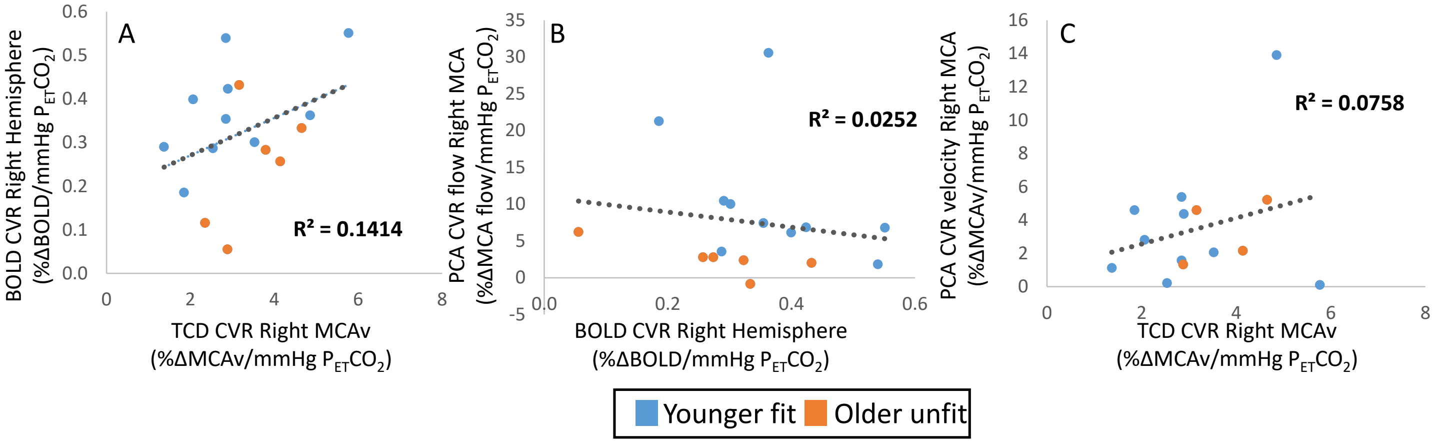

Figure S7: *Study 2:* Shows the correlation of CVR measures for the *right hemisphere only*, derived from: (**A**) TCD and BOLD measures, (**B**) BOLD and PCA MCA flow measures and (**C**) TCD MCA velocity and PCA MCA velocity measures. No significant correlations over the whole cohort were found.

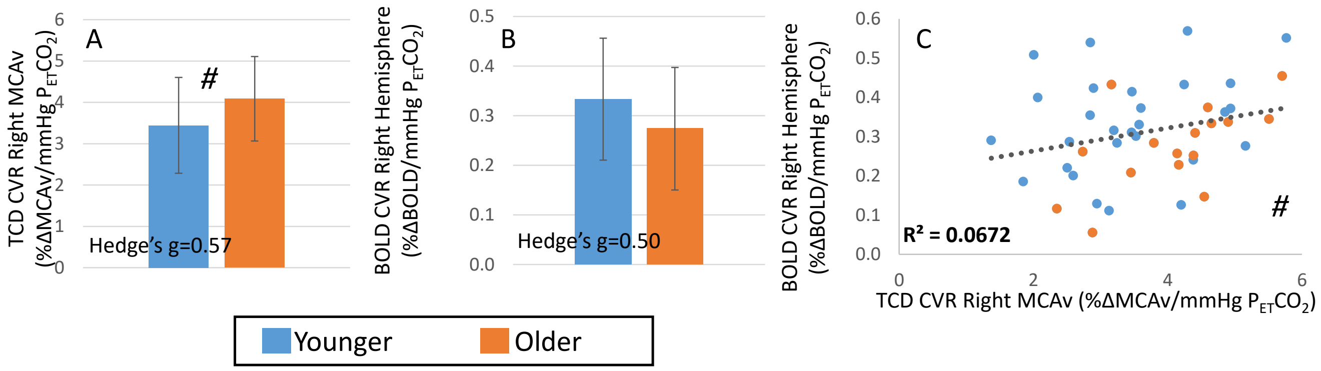

Figure S8: Considering the *right hemisphere only*. Comparison of CVR measures pooled over *Study 1 and 2* for with TCD (**A**) and BOLD (**B**) across younger (blue) and older (orange) groups. Error bars show the standard deviation across the group. # denotes a trend (p≤0.1, t-test) between groups. **C** shows the correlation of CVR measures from BOLD and TCD data, a trend (p≤0.1) in the correlation between measures was seen over the whole cohort.
